# Clinical impact of plasma cell-free DNA metagenomic next-generation sequencing testing in neonatal and infant populations

**DOI:** 10.1017/ash.2026.10774

**Published:** 2026-07-06

**Authors:** Neema Pithia, Kalpashri Kesavan, Annabelle Lee, Shangxin Yang, Ishminder Kaur

**Affiliations:** UCLA: University of California, Los Angeles, USA

## Abstract

**Objective:**

Plasma cell-free DNA metagenomic next-generation sequencing (cf-mNGS) tests offer the ability to detect microbial DNA from a single blood sample; however, its clinical utility in infants remains incompletely characterized. This study aims to evaluate the real-world clinical impact of plasma cf-mNGS testing in the neonatal and infant population.

**Design:**

Retrospective cohort study

**Setting:**

A large academic medical center in Los Angeles, California

**Patients:**

95 hospitalized neonates and infants (≤12 months of age).

**Methods:**

Clinical impact was adjudicated using predefined criteria.

**Results:**

We reviewed 95 unique plasma cf-mNGS testing episodes performed between February 2018 and August 2024. The mean age at testing was 4.2 months (SD, 3.8). All patients were hospitalized in the intensive care unit at the time of testing. Tests were most frequently performed for evaluation of “culture-negative sepsis” (30.5%), unexplained hospital-onset fevers (25.3%), and multiorgan failure (21.1%). Plasma cf-mNGS testing did not influence clinical management in the majority of cases (86.3%; 95% CI, 78.0%–91.8%). Positive clinical impact occurred in 5/95 cases (5.3%; 95% CI, 2.3%–11.7%), where plasma mNGS results assisting in antimicrobial de-escalation/discontinuation or earlier/new diagnoses. Negative clinical impact occurred in 4/95 cases (4.2%; 95% CI, 1.6%–10.3%), with plasma cf-mNGS results prompting unnecessary investigations or treatment.

**Conclusions:**

Our findings do not support the routine use of plasma cf-mNGS testing for indications including “culture-negative sepsis” in neonatal and infant populations.

## Introduction

Early and accurate pathogen identification is the cornerstone of effective care for neonates and infants with suspected infection. The increased sensitivity of modern blood culture systems to identify even low colony-count bacteremia coupled with optimization of blood culture collection techniques and target volumes can effectively mitigate several reservations regarding the diagnostic performance of blood cultures, particularly in the context of prior antibiotic exposure.^1^ The need for advanced infectious disease (ID) diagnostics remains in this patient population given the frequency of sepsis evaluations, which coupled with inadequate blood culture volume and clinician concern for missed detections on routine blood cultures, frequently results in the practice of treating “culture-negative sepsis,” often with broad-spectrum antibiotics.

Plasma cell-free DNA metagenomic next-generation sequencing (cf-mNGS) assays offer broad, noninvasive pathogen detection from a single blood sample, but interpretation is challenging as detected microbial DNA may reflect colonization, translocation, or clinically insignificant signal rather than true infection.^2–8^ Data on the real-world clinical impact of plasma cf-mNGS testing among infants has been explored in other countries, but data for its application using the commercially available test in North American institutions remains largely unexplored.^9–11^

This study aims to evaluate the real-world clinical impact of plasma cf-mNGS testing in the neonatal and infant population. Our secondary objective was to focus on the clinical performance of the test when sent for the diagnostic indication of “culture-negative sepsis”.

## Methods

### Study design

This study stemmed from a previously published retrospective study of plasma cf-mNGS testing in a large cohort of adult and pediatric patients performed between 2017 and 2023 at a large academic medical center in Los Angeles, California.^8^ We noted in our original study that a substantial number of plasma cf-mNGS tests were sent among hospitalized infants (≤12 months). This study focuses specifically on the unique clinical context of ID diagnostics in infants, with an expanded cohort from February 2018 through August 2024. The data for this cohort was re-abstracted and re-adjudicated to ensure accuracy and context-specific clinical impact assessment. If multiple tests were performed for a single patient during the study period, only the initial test was included to minimize misclassification of a single, ongoing illness as multiple infectious episodes.

The plasma cf-mNGS testing episodes were assigned for clinical adjudication to 4 investigators: ID specialist, neonatologist, a dual specialist in ID and neonatology, and a clinical microbiologist. Data was abstracted and initial clinical impact assigned by one of the investigators (NP). Each case was then reviewed by two other investigators for data accuracy and clinical impact adjudication. The clinical microbiologist was engaged to review and resolve any discrepancies in clinical adjudication. Plasma cf-mNGS testing in our cohort was performed by Karius Inc. (Redwood City, California). The minimum recommended blood volume requirements for blood culture and plasma cf-mNGS are 1 mL and 0.7 mL, respectively.

Patient charts were reviewed in the electronic medical record system to collect demographics, comorbidities, diagnostic indications, conventional microbiologic tests, provider documentation pertaining to plasma cf-mNGS test results, and patient location. Patient comorbidities and diagnostic indications were assigned to predefined categories (Supplementary Tables 1 and 2). Each patient was assigned a maximum of 2 diagnostic indication categories. Clinical impact was adjudicated using definitions in our previously published work8.^8^ Plasma cf-mNGS missing a pathogen detected by conventional microbiologic testing was categorized as neutral clinical impact, consistent with our original study. This classification reflects the understanding that plasma cf-mNGS generally has lower sensitivity than targeted microbiological tests for specific pathogens.

Diagnostic stewardship of plasma cf-mNGS testing at our institution was implemented in a stepwise fashion during the study period. Before August 2018, test ordering occurred without formal oversight. Between August 2018 and February 2023, each order underwent manual review by microbiology postdoctoral staff to ensure approval by an ID specialist. After February 2023, ordering was further restricted through computerized provider order entry to ID physicians only.

### Statistical approach

Descriptive statistics were computed for demographic characteristics and clinical impact, including means with standard deviation and percentages where appropriate. Proportions and two-sided 95% confidence intervals were calculated using the Wilson score method for binomial proportions. Because the diagnostic categories were not mutually exclusive, the percentages reported may not total 100%. All statistical analyses were conducted using R version 4.3.2.

This study was reviewed by the UCLA Human Research Protection Program and received an Institutional Review Board exemption.

## Results

### Demographics and plasma cf-mNGS results summary

The study included 103 plasma cf-mNGS tests performed between February 2018 and August 2024. After excluding 5 duplicate tests in the same patients and 3 tests not performed due to technical issues, 95 unique patients were included, including 15 new patients which were not included in our original study. The cohort had a mean age of 4.2 months (SD, 3.8). At the time of plasma cf-mNGS testing, all patients were cared for in intensive care settings, with 59 of 95 patients (62.1%) admitted to the combined pediatric or cardiothoracic intensive care unit (PICU/PCTU), while the remaining patients were in the neonatal ICU (NICU). Baseline demographic characteristics, comorbidities, care setting, and diagnostic indications are summarized in Table 1.

**Table 1.** Baseline characteristics, care setting, and diagnostic indications for plasma cell-free DNA metagenomic next-generation sequencing (cf-mNGS) testing in neonates and infants (n = 95)

Demographics	n (%)
Age at plasma cf-mNGS testing, months mean, (SD)	4.2 (3.8)^*^
Sex	
Female	52 (54.7%)
Male	43 (45.3%)
Gestational age	
22–24	5 (5.3%)
24–28	5 (5.3%)
28–32	1 (1.1%)
32–34	6 (6.3%)
34–37	22 (23.2%)
37+	56 (58.9%)
Birth weight categories	
ELBW	10 (10.5%)
LBW	15 (15.8%)
VLBW	1 (1.1%)
**Comorbidities** ^†^	
Complex congenital cardiac disease	32 (33.7%)
Congenital anomaly	4 (4.2%)
Genetic anomaly	2 (2.1%)
HIE	2 (2.1%)
Hydrops	2 (2.1%)
Inborn errors of metabolism	4 (4.2%)
Liver failure	4 (4.2%)
Post liver transplant	6 (6.3%)
Oncology diagnosis	1 (1.1%)
Primary immunodeficiency	4 (4.2%)
Renal anomalies	2 (2.1%)
Prematurity	39 (41.1%)
Prematurity without other comorbidities	9 (9.5%)
None documented	23 (24.2%)
**Patient Location at the time of plasma cf-mNGS testing**	
Pediatric ICU or cardiothoracic ICU	59 (62.1%)
Neonatal ICU	36 (37.9%)
**Diagnostic indications** ^‡^	
Invasive fungal infection	6 (6.3%)
Musculoskeletal Infections	4 (4.2%)
Multiorgan failure	20 (21.1%)
Culture negative sepsis	29 (30.5%)
Meningoencephalitis	11 (11.6%)
CNS infection including lesions (not abscess)	1 (1.1%)
Deep seated abscesses (liver abscess or intra-abdominal abscess)	7 (7.4%)
Acute liver failure or acute hepatitis	2 (2.1%)
Pneumonia in immunocompromised patient	4 (4.2%)
Complicated pneumonia in immunocompetent patients	1 (1.1%)
Culture negative Endocarditis	4 (4.2%)
Concern for Fastidious organisms (like Bartonella, Rickettsia, Q fever) or zoonotic/vector-borne pathogens	2 (2.1%)
Febrile neutropenia	1 (1.1%)
Unexplained hospital-onset fevers	24 (25.3%)
Skin and soft tissue infections, including rashes	6 (6.3%)
Congenital infection workup	1 (1.1%)

* Mean (standard deviation).

† Comorbidities not mutually exclusive.

‡ Diagnostic indications not mutually exclusive.

ELBW, extremely low birth weight; LBW, low birth weight; VLBW, very low birth weight; ICU, intensive care unit.

The most common comorbidity was complex congenital cardiac disease (n = 32, 33.7%). Prematurity was also frequent (n = 39, 41.1%); notably prematurity in the absence of other major comorbid conditions remained a prominent comorbidity (n = 9, 9.5%). Additional comorbidities are shown in Table 1.

Plasma cf-mNGS testing was most frequently obtained for evaluation of “culture-negative sepsis” (n = 29, 30.5%), unexplained hospital-onset fevers (n = 24, 25.3%), and multiorgan failure (n = 20, 21.1%). Central nervous system (CNS) infections were also a common indication, with testing sent for meningoencephalitis in 11 patients (11.6%) and for a spinal abscess in 1 patient (1.1%). Among NICU patients, evaluations were evenly split between early-onset sepsis (EOS; n = 18, 18.9%) and late-onset sepsis (LOS; n = 18, 18.9%). Other less frequent indications are shown in Table 1.

### Clinical impact assessment

Plasma cf-mNGS testing had a predominantly neutral impact in this cohort, with 82 of 95 tests (86.3%; 95% CI, 78.0%–91.8%) categorized as having neutral impact. Most neutral impact results were not acted upon by the clinical team (n = 53, 55.8%); the remainder included confirmation of a known diagnosis or clinical suspicion with continuation of the existing management plan (n = 27, 28.4%) and failure to detect a causative pathogen identified by conventional microbiologic testing (n = 2, 2.1%). In the latter cases, *Candida metapsilosis* was isolated from a mediastinal wound culture obtained one day later in one patient, and *Staphylococcus aureus* was isolated from a lymph node incision and drainage specimen obtained one day later in another patient. In 4/95 cases (4.2%; 95% CI, 1.6%–10.3%), clinical impact could not be determined from chart review (Supplementary Table 3).

Negative clinical impact was observed in 4/95 cases (4.2%; 95% CI, 1.6%–10.3%), most commonly when the cf-mNGS results prompted additional diagnostic investigations deemed unnecessary (n = 3, 3.2%) or contributed to unnecessary antimicrobial therapy (n = 1, 1.1%). Positive clinical impact was identified in 5/95 cases (5.3%; 95% CI, 2.3%–11.7%), including antimicrobial de-escalation or discontinuation of therapy (n = 3, 3.2%), earlier diagnosis than achieved by conventional microbiologic testing (n = 1, 1.1%; incidental identification of cytomegalovirus in a preterm infant which was confirmed by dedicated testing), and identification of pathogens not confirmed by conventional testing (n = 1, 1.1%, identification of *Haemophilus influenzae* as the cause of meningoencephalitis, which was subsequently confirmed by cerebrospinal fluid cf-mNGS testing) (Table 2).

**Table 2.** Clinical impact breakdown

Clinical Impact Breakdown *(n = 95)*	n (%)	#/total (%)
**Neutral**		82/95 (86.3)
Result not acted upon by clinical team	53 (55.8)	
Karius result confirmed clinical suspicion that enabled providers to continue management plan	9 (9.5)	
Karius result confirmed known diagnosis which enabled providers to continue the same management plan	18 (18.9)	
Failure to detect causative pathogen identified by conventional microbiologic testing	2 (2.1)	
**Indeterminate**		4/95 (4.2)
Could not determine clinical impact from chart review	4 (4.2)	
**Negative/Futile**		4/95 (4.2)
Karius result led to additional diagnostic investigations that were considered unnecessary or led to negative change in management	3 (3.2)	
Karius result led to unnecessary treatment per infectious diseases team involved	1 (1.1)	
**Positive**		5/95 (5.3)
Karius result enabled de-escalation or discontinuation of therapy	3 (3.2)	
New diagnosis made earlier by Karius result than conventional microbiological methods	1 (1.1)	
New diagnosis not confirmed by conventional microbiological methods	1 (1.1)	

Positive clinical impact differed significantly across age groups when compared with our prior publication (8), occurring in 5 of 95 infant tests (5.3%) in this study and 33 of 219 pediatric tests (>1 years <18) (15.1%) and 120 of 701 adult tests (17.1%) in the prior study (overall χ^2^ test, *P* = .01). In pairwise comparisons with Holm adjustment for multiple testing, the infant group had a significantly lower rate of positive clinical impact than both the pediatric group (5.3% vs 15.1%, adjusted *P* = .03), and the adult group (5.3% vs 17.1%, adjusted *P* = .01), whereas the difference between the pediatric and adult groups was not statistically significant (15.1% vs 17.1%, adjusted *P* = .48).

### “Culture-negative sepsis” cohort (n = 29)

Plasma cf-mNGS was performed for the indication of culture-negative sepsis in the first 6 weeks of life in 21/29 infants (mean age 18 days, SD 14) and at >3 months of age in the remainder 8/29 infants (mean age 262 days). Clinical impact was neutral with results not acted upon in the vast majority (25/29, 86%), with positive clinical impact adjudicated in 2 cases where it helped providers de-escalate antimicrobial therapy, and negative in 2 cases where it led to additional investigations deemed unnecessary (Supplementary Table 3). Plasma cf-mNGS test found no organisms in 12 cases and a range of organisms (1–6) in other cases, the majority (37/40, 93%) of which were deemed false positives, and the rest being previously known results by conventional microbiological testing (Supplementary Table 4).

## Discussion

In this single-center retrospective study of neonates and infants hospitalized in intensive care settings, plasma cf-mNGS testing demonstrated limited real-world clinical impact. Most tests had neutral impact (86.3%), whereas positive, negative, and indeterminate impact were uncommon (5.3%, 4.2%, and 4.2%, respectively). These findings suggest that plasma cf-mNGS is not consistently additive to conventional diagnostic approaches and infrequently changes clinical management in this high-acuity population. The positive clinical impact of plasma cf-mNGS testing was significantly lower in the infant population than in our prior published pediatric and adult populations. As shown in our original study, the clinical impact of the test was predicted better by diagnostic indications than host comorbidities. We hypothesize that the predominance of “culture-negative sepsis”, multiorgan failure, and unexplained hospital-onset fevers as test indications in the infant cohort may have contributed to the lower positive clinical impact, as these indications were associated with lower positive clinical impact in the pairwise univariable analysis performed in the original study.^8^ The practical constraints of neonatal care further temper enthusiasm for broad use: obtaining adequate blood volume is challenging and iatrogenic phlebotomy is a meaningful consideration, particularly in premature or medically fragile infants with limited physiological reserve.

There is considerable interest in advanced ID diagnostics to optimize care of children treated with antibiotics for “culture-negative sepsis.” However, a recent prospective study found detectable plasma cell-free microbial DNA in 10 of 20 well-appearing hospitalized preterm neonates, raising concerns about limited specificity in this setting.^11^ Our study confirms this limited utility in real-world practice. Plasma cf-mNGS testing performed for the indication of culture-negative sepsis found a range of pathogens (1–6) in 60% (17/29) of cases. Apart from 3 pathogens, which were already known by blood cultures, the majority of the organisms detected by plasma cf-mNGS were considered clinically insignificant. The latter included several organisms of pathogenic potential, including *Escherichia coli*, *Aspergillu*s spp., *Candida* spp., *Enterococcus faecalis,* etc., which highlights the degree of clinical correlation needed for plasma cf-mNGS test result interpretation.

Several limitations should be considered when interpreting these findings. This study reflects practice at a single academic institution with a heterogenous cohort with a high level of complex medical and surgical care, which may influence pretest probability, competing diagnostic data streams, and provider-specific thresholds for acting on results, which thereby limits generalizability. A substantial proportion of patients in each impact category had comorbidity status recorded as unknown, constraining inference regarding associations between comorbidity burden and test-related clinical impact. The timing of plasma cf-mNGS testing relative to symptom onset, antimicrobial initiation, and collection of conventional microbiologic studies was variable in this cohort, which may have influenced diagnostic yield and downstream clinical impact. Although antibiotic exposure and timing of plasma cf-mNGS could provide valuable information on test utility, the acuity of this cohort and the heterogeneity in antimicrobial initiation relative to cf-mNGS sampling limited reliable interpretation. Future prospective studies in more uniform populations with standardized sampling relative to antimicrobial exposure are needed. Finally, retrospective assessment of “impact” is inherently dependent on documentation and may underestimate subtle influences on decision-making.

Overall, plasma cf-mNGS should be used judiciously in hospitalized critically ill neonates and infants: it is not a panacea, and conventional microbiology, syndrome-driven testing, and clinical reasoning remain the cornerstone of infectious disease evaluation in this population. Future prospective, multicenter studies should focus on defining high-yield clinical scenarios, optimizing timing relative to antibiotics and other diagnostics, investigating larger homogenous cohorts, and integrating plasma cf-mNGS into structured interpretation pathways aligned with antimicrobial stewardship principles to maximize benefit while reducing unnecessary downstream testing and treatment.

## Supporting information

10.1017/ash.2026.10774.sm001 Pithia et al. supplementary material Pithia et al. supplementary material

## Supplementary material

The supplementary material for this article can be found at https://doi.org/10.1017/ash.2026.10774.
