# Supplementary material for: Clinical impact of plasma cell-free DNA metagenomic next-generation sequencing testing in neonatal and infant populations
Source: Antimicrob Steward Healthc Epidemiol. 2026 Jul 6;6(1):e201. doi: 10.1017/ash.2026.10774 (PMC13343332; doi:10.1017/ash.2026.10774)
Supplement: Pithia et al. supplementary material [file S2732494X26107748sup001.docx]

**Supplementary Table 1.** Comorbidities Categorization for Plasma cell-free DNA Metagenomic Next-Generation Sequencing Testing Clinical Impact

| **Patient Comorbidity Categories** |
| --- |
| Complex Congenital Cardiac Disease |
| Congenital Anomaly |
| Genetic Anomaly |
| HIE |
| Hydrops |
| Inborn Errors of Metabolism |
| Liver failure |
| Post Liver Transplant |
| Oncology Diagnosis |
| Primary immunodeficiency |
| Renal anomalies |
| Prematurity |
| Unknown |

**Supplementary Table 2. Diagnostic Indication Categories for Plasma cell-free DNA Metagenomic Next-generation Sequencing Testing Clinical Impact**

| Invasive Fungal Infection |
| --- |
| Musculoskeletal Infections |
| Multiorgan failure |
| Culture negative sepsis |
| Meningoencephalitis |
| CNS infection including lesions (not abscess) |
| Deep seated abscesses (liver abscess or intra-abdominal abscess) |
| Acute liver failure or acute hepatitis |
| Pneumonia in immunocompromised patient |
| Complicated pneumonia in immunocompetent patients |
| Culture negative Endocarditis |
| Concern for Fastidious organisms (like Bartonella, Rickettsia, Q fever) or zoonotic/vector-borne pathogens |
| Febrile neutropenia |
| Unexplained hospital-onset fevers |
| Skin and soft tissue infections, including rashes |
| Congenital infection workup |

**Supplementary Table 3. Plasma cell-free DNA metagenomic next-generation sequencing assay clinical impact by co-morbidities and diagnostic indications.**

| **Co-morbidities** | **Negative** (n=6) | **Neutral/None** (n=84) | **Positive** (n=5) |
| --- | --- | --- | --- |
| Complex Congenital Cardiac Disease | 3 (50%) | 26 (31%) | 3 (60%) |
| Congenital Anomaly | 0 (0%) | 4 (4.8%) | 0 (0%) |
| Genetic Anomaly | 0 (0%) | 2 (2.4%) | 0 (0%) |
| Hypoxic Ischemic Encephalopathy (HIE) | 0 (0%) | 2 (2.4%) | 0 (0%) |
| Hydrops | 0 (0%) | 2 (2.4%) | 0 (0%) |
| Inborn Errors of Metabolism | 1 (16.7%) | 3 (3.6%) | 0 (0%) |
| Liver failure | 0 (0%) | 4 (4.8%) | 0 (0%) |
| Post Liver Transplant | 0 (0%) | 6 (7.1%) | 0 (0%) |
| Oncologic Diagnosis | 0 (0%) | 1 (1.2%) | 0 (0%) |
| Primary immunodeficiency | 0 (0%) | 4 (4.8%) | 0 (0%) |
| Renal anomalies | 0 (0%) | 2 (2.4%) | 0 (0%) |
| Prematurity alone | 2 (33.3%) | 33 (39.2%) | 2 (40%) |
| Unknown | 0 (0%) | 28 (33%) | 0 (0%) |
| **Diagnostic Indication** | **Negative** (n=6) | **Neutral/None** (n=84) | **Positive** (n=5) |
| Invasive Fungal Infection | 0 (0%) | 6 (7.1%) | 0 (0%) |
| Musculoskeletal Infections | 0 (0%) | 4 (4.8%) | 0 (0%) |
| Multiorgan failure | 3 (50%) | 14 (16.7%) | 3 (60%) |
| Culture negative sepsis | 2 (33.3%) | 25 (29.8%) | 2 (40%) |
| Meningoencephalitis | 1 (16.7%) | 9 (10.7%) | 1 (20%) |
| CNS infection including lesions (not abscess) | 0 (0%) | 1 (1.2%) | 0 (0%) |
| Deep seated abscesses (liver abscess or intra-abdominal abscess) | 0 (0%) | 7 (8.3%) | 0 (0%) |
| Acute liver failure or acute hepatitis | 0 (0%) | 2 (2.4%) | 0 (0%) |
| Pneumonia in immunocompromised patient | 0 (0%) | 4 (4.8%) | 0 (0%) |
| Complicated pneumonia in immunocompetent patients | 0 (0%) | 1 (1.2%) | 0 (0%) |
| Culture negative Endocarditis | 1 (16.7%) | 2 (2.4%) | 1 (20%) |
| Concern for Fastidious organisms (like Bartonella, Rickettsia, Q fever) or zoonotic/vector-borne pathogens | 1 (16.7%) | 1 (1.2%) | 0 (0%) |
| Febrile neutropenia | 0 (0%) | 1 (1.2%) | 0 (0%) |
| Unexplained hospital-onset fevers | 0 (0%) | 22 (26.2%) | 2 (40%) |
| Skin and soft tissue infections, including rashes | 1 (16.7%) | 5 (6%) | 0 (0%) |
| Congenital infection workup | 0 (0%) | 1 (1.2%) | 0 (0%) |

**Supplementary Table 4. Clinical details for cases where plasma cell-free DNA metagenomic next-generation sequencing (cf-mNGS) testing performed for diagnostic indication of culture-negative sepsis.**

| Patient number | Gestational age | Age at the time of plasma cf-mNGS testing, days | Number of organisms detected by plasma cf-mNGS test | Organisms detected by plasma cf-mNGS | Clinical Impact of plasma cf-mNGS test | Clinical details, when plasma cf-mNGS detected organisms or results were acted upon |
| --- | --- | --- | --- | --- | --- | --- |
| 1 | 33 weeks | 14 | 0 | none | Neutral | -- |
| 2 | 27 weeks | 41 | 5 | *Candida parapsilosis, Mycoplasma cynos, Enterococcus faecalis, Klebsiella pneumonaie, Mycobacterium fortuitum group* | Neutral | Patient with known candidemia, rest deemed clinically insignificant |
| 3 | 41 weeks | 6 | 1 | *Peptoniphilus harei* | Neutral | Deemed clinically insignificant |
| 4 | 25 weeks | 8 | 3 | *Eschericia coli, Candida albicans, Staphyloccocus epidermidis* | Neutral | Patient with known candidemia, rest deemed clinically insignificant |
| 5 | 28 weeks | 3 | 6 | *Achromobacter ruidilans, Achromobacter xyloxidans, Klebsiella michiganesis, Pseudomonas fluoresnces, Snethia sanguinous, Fusobacterium nucleatum* | Neutral | Deemed clinically insignificant |
| 6 | 24 weeks | 20 | 1 | *Enterococcus faecalis* | Neutral | Deemed clinically insignificant |
| 7 | 35 weeks | 46 | 2 | *Prevotella meningiogenica,Eschericia coli,* | Neutral | Deemed clinically insignificant |
| 8 | 38 weeks | 16 | 1 | *Pseudomonas aeruginosa* | Neutral | Deemed clinically insignificant |
| 9 | 38 weeks | 111 | 3 | *Candida tropicalis, Aspergillus oryzae, Aspergillus flavus* | Neutral | Initially treated for aspergillosis but clinical picture not consistent, ultimately deemed clinically insignificant |
| 10 | 39 weeks | 330 | 0 | none | Neutral | -- |
| 11 | 38 weeks | 224 | 2 | HHV6B, *Trichoderma atroviride* | Neutral | Deemed clinically insignificant |
| 12 | 37 weeks | 24 | 1 | *Staphylococcus epidermidis* | Positive | Confirmed S. epidermidis as only pathogen and enabled de-escalation |
| 13 | 35 weeks | 42 | 2 | *Pseudomonas aeruginosa, Enterobacer cloaecae complex* | Neutral | Deemed clinically insignificant |
| 14 | 38 weeks | 4 | 2 | *Acinetobacter haemolyticus, Psuedomonas pseudoalcaligenes* | Neutral | Deemed clinically insignificant |
| 15 | 40 weeks | 26 | 0 | none | Neutral | -- |
| 16 | 35 weeks | 38 | 0 | none | Neutral | -- |
| 17 | 39 weeks | 7 | 0 | none | Neutral | -- |
| 18 | 39 weeks | 5 | 0 | none | Neutral | -- |
| 19 | 35 weeks | 18 | 0 | none | Neutral | --- |
| 20 | 40 weeks | 338 | 1 | HHV6 | Negative | Led to unnecessary diagnostic tests |
| 21 | 35 weeks | 14 | 0 | none | Neutral | -- |
| 22 | 35 weeks | 8 | 0 | none | Neutral | -- |
| 23 | 35 weeks | 346 | 1 | EBV | Negative | Led to unnecessary diagnostic tests |
| 24 | 39 weeks | 103 | 3 | *Klebisiella pneumonia, Acinetbacter haemolytics, Bacteroides diastases* | Neutral | Deemed clinically insignificant |
| 25 | 39 weeks | 4 | 4 | *Streptococcus thermophilis, Acinetobacter haemolyticus* | Neutral | Deemed clinically insignificant |
| 26 | 39 weeks | 374 | 0 | none | Neutral | -- |
| 27 | 39 weeks | 7 | 2 | *Eschericia coli, Weeksela virosa* | Neutral | Deemed clinically insignificant |
| 28 | 34 weeks | 275 | 0 | none | Neutral | -- |
| 29 | 39 weeks | 39 | 0 | none | Positive | Negative results enabled antibiotic de-escalation |
